# Supplementary material for: Distinct thresholds condition sense posttranscriptional gene silencing initiation and amplification
Source: Plant Cell. 2026 Jun 9;38(6):koag166. doi: 10.1093/plcell/koag166 (PMC13318159; doi:10.1093/plcell/koag166)
Supplement: koag166_Supplementary_Data [file koag166_supplementary_data.zip › 2604015-sup-Figures.pdf]

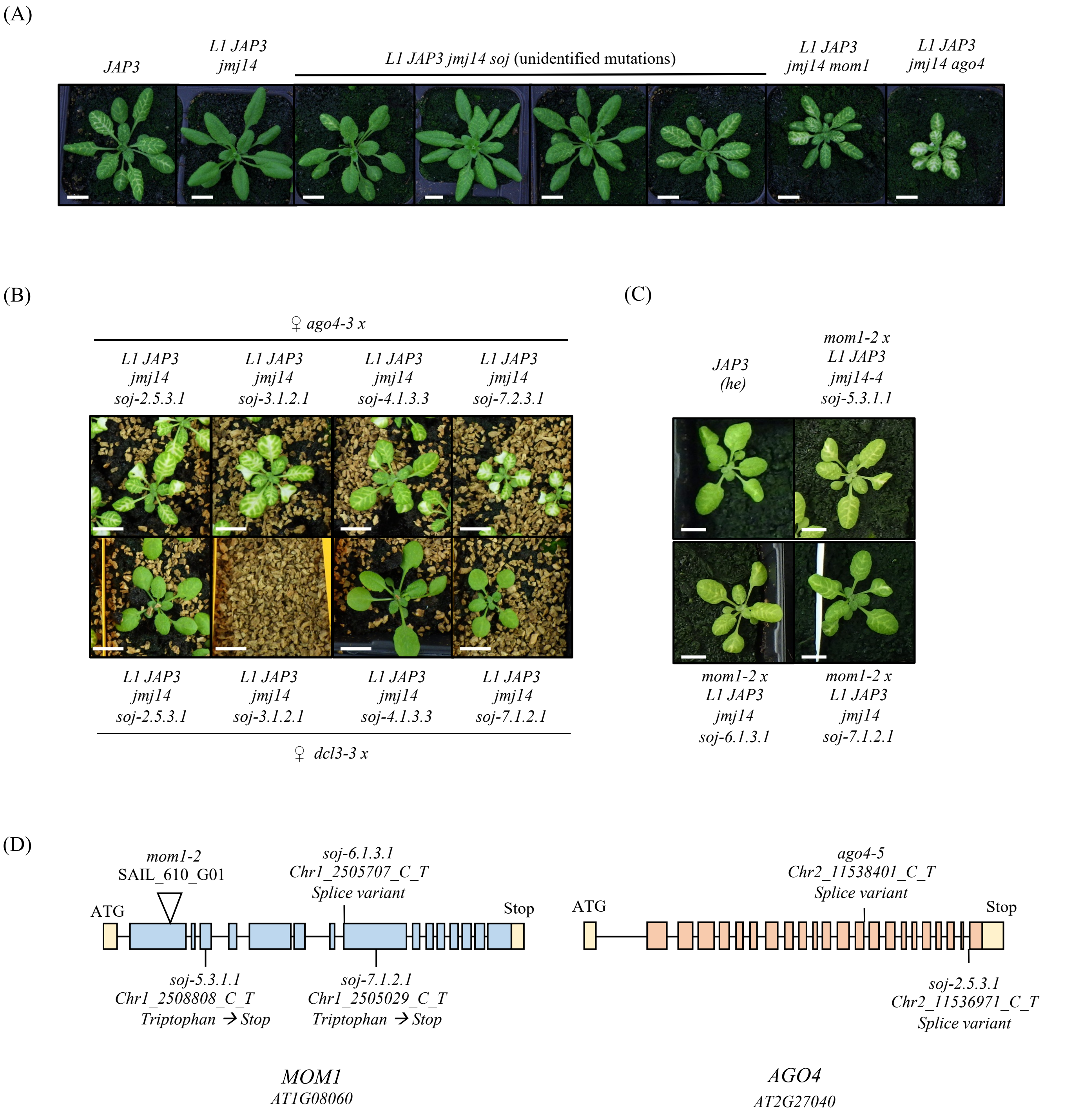

Supplemental Figure 1: Mutations in *AGO4* and *MOM1* suppress the effect of *jmj14* on *JAP3* silencing

(A) Range of phenotypes of suppressor mutants isolated from the mutagenesis of the *L1 JAP3 jmj14* line. *L1 JAP3 jmj14 ago4* and *L1 JAP3 jmj14 mom1* exhibit the higher levels of photobleaching. Scale bars, 1cm.

(B, C) Complementation assay of the different *L1 JAP3 jmj14 ago4* (B) and *L1 JAP3 jmj14 mom1* (C) mutants. Control crosses with *dcl3* mutants (B) or heterozygous *JAP3* lines (C) shows the silenced *JAP3* phenotype. Scale bars, 1cm.

(D) Schematic representation of *AGO4* and *MOM1* including the localization of the different *soj* mutations identified in this study. The filled squares correspond to exons.

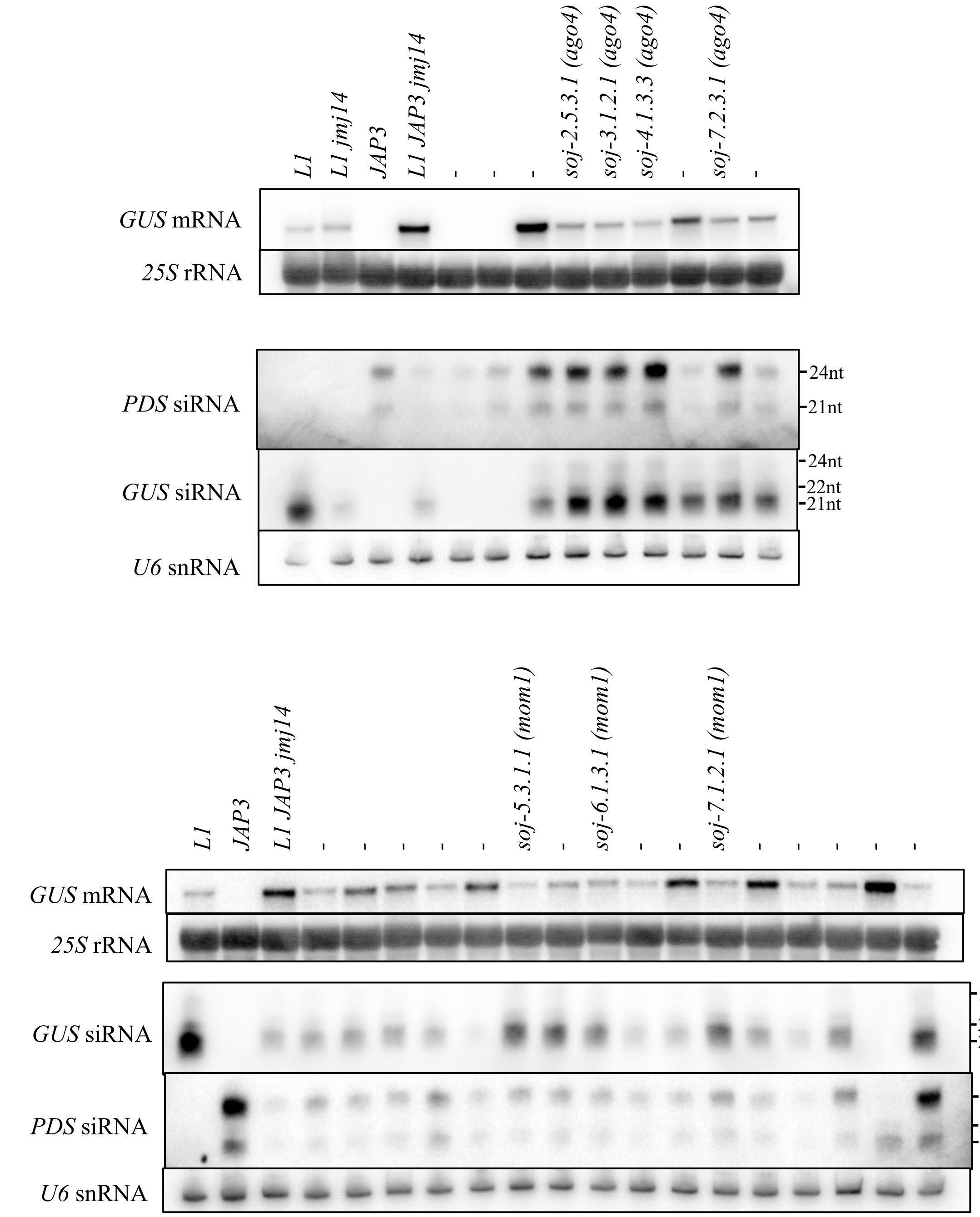

Supplemental Figure 2: Original blots of the Figure 2

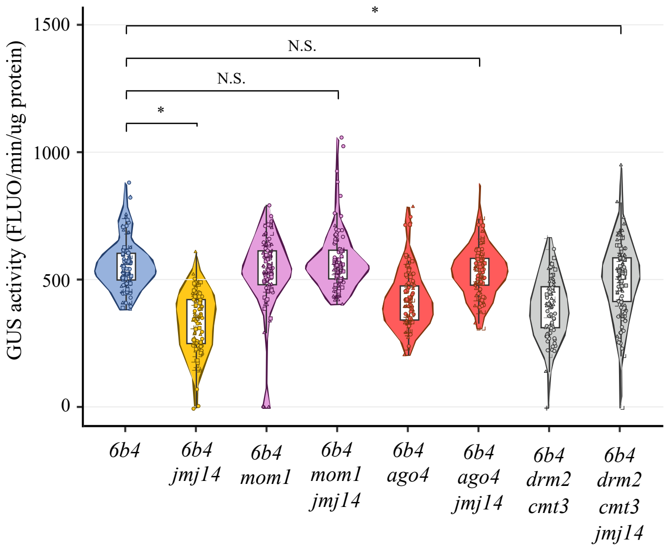

Supplemental Figure 3 : Mutations in *AGO4, MOM1* and *DRM2/CMT3* restore *6b4* GUS activity at wildtype levels in a *JMJ14*-deficient background

GUS activity in aerial parts of 14 days after germination measured in *6b4* and *6b4 jmj14* plants carrying second site mutations (*ago4*, *mom1* and *drm2 cmt3*) suppressing the effect of *jmj14*. Each dot represents an individual plant, with more than 80 plants tested per genotype. P-values were calculated using non parametric Wilcoxon tests adjusted with Holm–Bonferroni correction (* means P<0.05 and N.S., not significant).

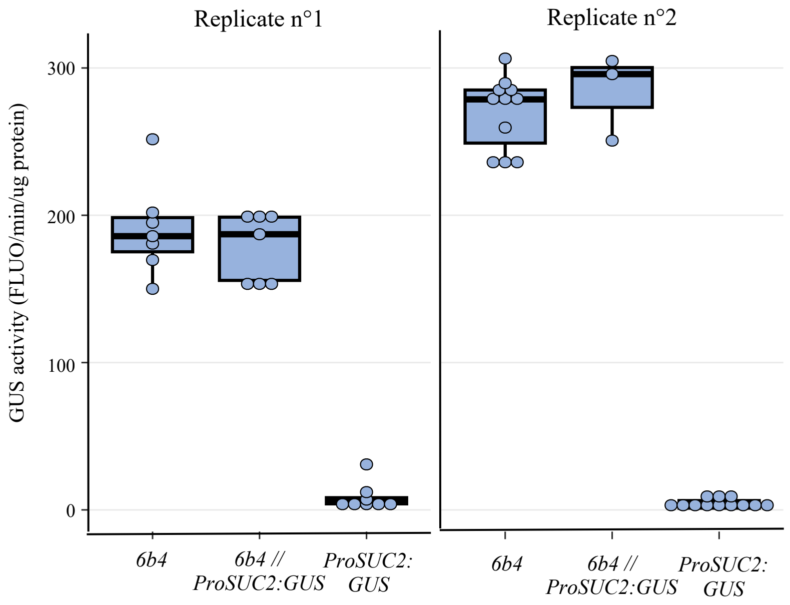

Supplemental Figure 4 : The *ProSUC2:GUS* line does not produce a systemic silencing signal

GUS activity was measured in leaves of *6b4*, *ProSUC2:GUS* and *6b4* plants grafted onto the *ProSUC2:GUS* line (*6b4 // ProSUC2:GUS*). Two independent grafting experiments are shown as separated panels.

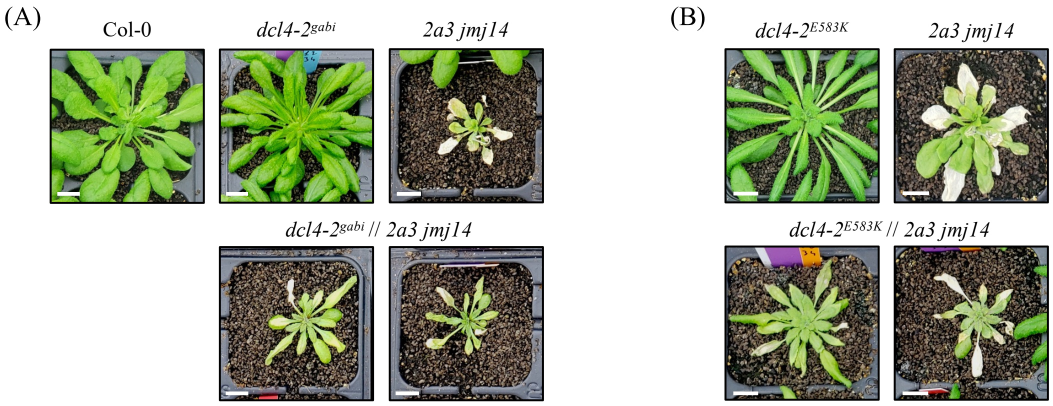

Supplemental Figure 5 : All *dcl4* alleles tested allow endogenous *NIAs* genes to trigger systemic PTGS upon grafting onto the *NIA* siRNA producing rootstock *2a3 jmj14* .

Images of aerial parts of distinct *dcl4* alleles grown on their own roots or grafted onto a silenced *Pro35S:NIA2* transgenic line (*2a3*) in a *jmj14* mutant background. (A) *dcl4-2*^{gabi}. (B) *dcl4-2*^{E583K}. *dcl4-5* is shown on Figure 5. Scale bars, 1cm.

No initiation / Systemic S-PTGS upon grafting onto a silenced roostock due to efficient amplification	Systemic S-PTGS due to spontaneous initiation and efficient amplification
6b4<br>*N11*<br>*6b4 ago4*<br>*6b4 mom1*<br>*6b4 jmj14 ago4*<br>*6b4 jmj14 mom1*<br>*endoNIA2 dcl4*	*L1*<br>*L2*<br>*L1 jmj14 ago4*<br>*L1 jmj14 mom1*<br>*6b4 iGUS* (after induction)<br>*ProSUC2:GUS 6b4*<br>*35S:NIA2*
N4<br>*6b4* (heterozygous)<br>*6b4 jmj14*<br>*ProSUC2:GUS*<br>*endoNIA2*	*L1 jmj14*
No S-PTGS at all<br>(no initiation / no amplification even upon grafting)	Spontaneous initiation but incomplete S-PTGS due to inefficient amplification

Supplemental Figure 6 : Initiation and amplification status of the different lines used in this study
